# Looking into the genetic bases of OCD dimensions: a pilot genome-wide association study

**DOI:** 10.1038/s41398-020-0804-z

**Published:** 2020-05-18

**Authors:** María Alemany-Navarro, Raquel Cruz, Eva Real, Cinto Segalàs, Sara Bertolín, Raquel Rabionet, Ángel Carracedo, Jose M. Menchón, Pino Alonso

**Affiliations:** 1 Institut d’Investigació Biomèdica de Bellvitge (IDIBELL), L’Hospitalet de Llobregat, Barcelona, Spain; 2 OCD Clinical and Research Unit, Psychiatry Department, Hospital Universitari de Bellvitge, Barcelona, Spain; 3 Department of Clinical Sciences, Bellvitge Campus, University of Barcelona, Barcelona, Spain; 4 Grupo de Medicina Xenómica, CIBERER, Centre for Research in Molecular Medicine and Chronic Diseases (CIMUS), Universidade de Santiago de Compostela, Santiago de Compostela, Spain; 5 CIBERSAM (Centro de Investigación en Red de Salud Mental), Instituto de Salud Carlos III, Madrid, Spain; 6 Institut de Recerca Sant Joan de Déu, Esplugues de Llobregat, Spain; 7 Institut de Biomedicina de la Universitat de Barcelona (IBUB), CIBERER, and Dept. Genetics, Microbiology & Statistics, Faculty of Biology, University of Barcelona, Barcelona, Spain; 8 Fundación Pública Galega de Medicina Xenómica, SERGAS, Instituto de Investigación Sanitaria de Santiago de Compostela (IDIS), Santiago de Compostela, Spain

**Keywords:** Diagnostic markers, Psychiatric disorders, Clinical genetics, Clinical genetics, Genomics

## Abstract

The multidimensional nature of obsessive-compulsive disorder (OCD) has been consistently reported. Clinical and biological characteristics have been associated with OCD dimensions in different ways. Studies suggest the existence of specific genetic bases for the different OCD dimensions. In this study, we analyze the genomic markers, genes, gene ontology and biological pathways associated with the presence of aggressive/checking, symmetry/order, contamination/cleaning, hoarding, and sexual/religious symptoms, as assessed via the Dimensional Yale-Brown Obsessive Compulsive Scale (DY-BOCS) in 399 probands. Logistic regression analyses were performed at the single-nucleotide polymorphism (SNP) level. Gene-based and enrichment analyses were carried out for common (SNPs) and rare variants. No SNP was associated with any dimension at a genome-wide level (*p* < 5 × 10^−8^). Gene-based analyses showed one gene to be associated with hoarding (*SETD3*, *p* = 1.89 × 10^−08^); a gene highly expressed in the brain and which plays a role in apoptotic processes and transcriptomic changes, and another gene associated with aggressive symptoms (*CPE*; *p* = 4.42 × 10^−6^), which is involved in neurotrophic functions and the synthesis of peptide hormones and neurotransmitters. Different pathways or biological processes were represented by genes associated with aggressive (zinc ion response and lipid metabolism), order (lipid metabolism), sexual/religious (G protein-mediated processes) and hoarding (metabolic processes and anion transport) symptoms after FDR correction; while no pathway was associated with contamination. Specific genomic bases were found for each dimension assessed, especially in the enrichment analyses. Further research with larger samples and different techniques, such as next-generation sequencing, are needed to better understand the differential genetics of OCD dimensions.

## Background

Obsessive-compulsive disorder (OCD) is a neuropsychiatric condition that has an estimated prevalence of 2–3%^1^. Despite the unitary nosological status of OCD (DSM-5), considerable heterogeneity of OCD symptoms exists. Several studies have looked into different symptom dimensions present in OCD; some have reported exploratory or confirmatory factor analysis based on Yale-Brown Obsessive Compulsive Scale Checklist (Y-BOCS-CL) items. Most of those studies have reported four or five main (second-order) factors, which in part depend on the methodology employed^2,3^, thereby suggesting a multidimensional model for OCD. This multidimensional nature of OCD has been confirmed by meta-analyses and systematic reviews^4,5^. Along these lines, specific instruments such as the Dimensional Yale-Brown Obsessive Compulsive Scale (DY-BOCS^6^;) have been developed to assess OCD severity in different symptom dimensions.

A range of clinical characteristics has been associated with OCD symptom dimensions in different ways. In this vein, it has been proposed that the hoarding and symmetry dimensions are characteristic of an early-age OCD group^7,8^. In terms of comorbidities, a factor comprising aggressive, sexual and religious symptoms has been associated with comorbid major depressive disorder and bipolar disorder (MDD/BD); while patients with symmetry/order symptoms show greater comorbidity with eating and addictive disorders as well as attention-deficit/hyperactivity disorder (ADHD)^9^. Meanwhile, the contamination/cleaning dimension has been reported as the dimension that is least frequently associated with any other Axis I disorder^10^. In addition, the hoarding and symmetry/order dimensions have been associated with a poorer response to pharmacological treatment^8,10^.

Differential endophenotypic profiles have also been reported in relation to symptom dimensions in neuroimaging studies. For instance, OCD probands with symmetry/order symptoms have been reported to present a reduced volume of the right precentral gyrus^11^ and the hippocampus, which is also associated with aggressive/checking symptoms when compared to healthy controls^12^. In addition, the aggressive/checking and contamination/cleaning dimensions have been negatively correlated to right cerebellum and right insula volumes, respectively^13^. In terms of functionality, patients with aggressive/checking and sexual/religious symptoms have been found to present greater amygdala activation when confronted by fear-inducing stimuli^14^. Also, differences in connectivity have been observed between the aggressive/checking, sexual/religious and hoarding dimensions^15^. Although we do not know to what extent these observed neurological differences between the OCD dimensions have a genetic basis, some of the structural and functional brain characteristics identified have been directly related to certain genetic variants in OCD patients as well as in those with other psychiatric disorders^16–20^.

In fact, specific genetic bases have been identified for the different OCD symptom dimensions. For instance, severity of the contamination/cleaning dimension has been associated with both the Met allele of the Vall66Met locus within the brain-derived neurotrophic factor gene (*BDNF*)^21^ and the c.256G allele of the 5-hydroxytryptamine receptor 3E (*HTR3E*) variant rs7627615^22^. The presence of this dimension has also been associated with the variants rs4657411 within the LIM homeobox transcription factor 1 alpha gene (*LMX1A*), and rs2075507 of the catechol-*O*-methyltranspherase gene (*COMT*) in women^23,24^. In addition, a protective role against these dimensions has been attributed to the ACCCG haplotype of the estrogen receptor 1 gene (*ESR1*)^25^. The symmetry/order dimension has been related to the S allele and the SS genotype of the serotonin transporter polymorphic region (SERTPR)^26^; the presence of the 2R allele within the dopamine receptor D4 gene (*DRD4*) 48-bp variable number of tandem repeats polymorphism (VNTR)^27^; and the A allele in the *COMT* variant rs2075507 in men^24^. This dimension in combination with aggressive/checking behavior has been associated with specific variants in a promoter region of the glutamate ionotropic receptor NMDA-type subunit 2B gene (*GRIN2B*) (rs1019385)^28^ and the SLIT and NTRK-like family member 1 gene (*SLITRK1)* (rs9593835); the latter, specifically in men^23^. The SERTPR has also been associated with higher scores in a religious/somatic dimension (l/s and l/l genotypes)^29^ and in counting and repeating rituals in OCD patients with a comorbid tic disorder (l/l genotype)^30^. Women who exhibit hoarding symptoms have been reported to present a higher frequency of the Met/Met genotype of the *COMT* variant rs4680 than those who do not. Hoarding has also been associated with a variant (rs1017412) within the neurotrophic receptor tyrosine kinase 3 gene (*NTRK*)^31^ and with both the short variant of the serotonin-transporter-linked polymorphic region (5HTTLPR), and its long variant together with the G allele at rs25531 in males^32^. A neutralization dimension, as assessed by the Obsessive-Compulsive Inventory-Revised (OCI-R) has been associated with a variant of *LMX1A* (rs4657411)^23^. Severity scores in a dimension comprising somatic and sensory phenomena symptoms have shown a trend towards an association with the Val58Met genotype of the *COMT* gene in interaction with sex, with women presenting lower scores^33^.

Although a large number of studies have focused on elucidating the genetic basis of OCD, inconsistent results have been reported in most respects^34^. A possible explanation for this is that the studies do not usually consider different symptom profiles among OCD patients. It has consistently been argued that it is necessary to account for OCD heterogeneity in genetic and neurobiological studies^35,36^. Therefore, in this study, we analyze the variants, genes and functional pathways that might be differentially involved in the OCD dimensions measured by the DY-BOCS^6^ through an exploratory genomic method. We hypothesize that different genomic bases will be found for the different OCD dimensions.

## Methods

### Subjects

Three hundred and ninety-nine Caucasian Spanish patients (*n* = 399; 210 women; mean age = 35 ± 11) with an OCD diagnosis were recruited from the OCD clinic at Bellvitge Hospital (Barcelona, Spain). Diagnoses were made by three psychiatrists with extensive clinical experience in OCD, following the DSM-IV criteria for OCD diagnosis^37^ using the Structured Clinical Interview for DSM-IV Axis I Disorders-Clinician Version (SCIDCV)^38^. All the patients had the disorder for at least one year. Those patients presenting psychoactive substance abuse/dependence (current or in the past six months), psychotic disorders, intellectual disability, severe organic or neurological pathology (except tic disorders), or autism spectrum disorders were excluded from the study. Other affective and anxiety disorders were not criteria for exclusion in cases where OCD was the main diagnosis.

Participants were required to give written consent after being fully informed about the study. The study was approved by the Ethical Committees of Bellvitge Hospital and was performed in accordance with the Helsinki Declaration of the World Medical Association.

### Clinical assessment

Medical data and both sociodemographic and clinical characteristics were collected via a structured interview during each patient’s first appointment at the clinic.

Age at onset was defined as the moment when obsessive symptoms reached a clinically significant level. Family psychiatric history was considered dichotomously, but specific information regarding family history of OCD, Tourette syndrome and depression was also collected. Only family members who had received a formal diagnosis were considered to be affected.

Baseline severity of obsessive and compulsive symptoms was also assessed through the clinician-administered version of the Y-BOCS^39^ during the patient’s first visit to the clinic. A global measure, as well as independent measures for both obsessions and compulsions, were recorded.

#### OCD Dimension: presence and severity

Dimension-specific presence and severity were evaluated using the DY-BOCS^6^, which is composed of a self-report part and a clinician-rated instrument. It assesses OCD severity in six different symptom dimensions that gather together thematically similar symptoms. The six are: aggressive obsessions and checking compulsions (aggressive/checking); symmetry obsessions and order compulsions (symmetry/order); contamination obsessions and cleaning/washing compulsions (contamination/cleaning); hoarding obsessions and compulsions (hoarding); sexual or religious obsessions accompanied by different rituals (sexual/religious); and a miscellaneous dimension including other obsessive thoughts and compulsive behavior (miscellaneous). We did not consider this last dimension in our analyses given its lack of specificity.

### Genotyping data and quality control

Our sample consisted of 399 OCD patients genotyped using the Infinium PsychArray-24 BeadChip from Illumina. This array was developed in collaboration with the Psychiatric Genomics Consortium (PGC) and includes 50,000 variants associated with common psychiatric disorders. Variant calling was performed using three different algorithms: GenCall, which is Illumina’s default calling algorithm, and Birdseed, both for common variants; and zCall, aimed at rare variant calling. A unique set of genotypes was derived from a consensus merge of the GenCall and Birdseed common variants, also including rare variants called by zCall that passed quality control (QC) from the consensus merge of GenCall and Birdseed.

QC filtering of genotype data was performed using PLINK^40^. Only non-monomorphic autosomal biallelic variants in Hardy Weinberg equilibrium (*p* < 0.0001) with a call rate of above 98% were included.

Samples that had a call rate lower than 98% were removed. Identity by descent was calculated using independent SNPs, and omitting those samples with a pi-hat greater than 0.2^41^. Population stratification was tested by principal component analysis, removing those samples that deviated by more than 5 standard deviations (SDs) from the mean in the first two components.

### Statistical analysis

Association analyses at the SNP level were performed using the GenABEL library for R software^42^. Regression analyses were carried out under a log-additive model (in which the genotypes were coded as 0, 1 or 2 depending on the number of minor alleles). These operations were performed for autosomal SNPs (markers showing a minor allele frequency (MAF) > 0.05 in autosomes). Given the non-normality of the DY-BOCS scores and the impossibility of normalizing them, these variables were dichotomized to analyze the presence/absence of the different DY-BOCS dimensions. A logistic regression analysis was performed for each dimension, with the dependent variable being the dimension we were testing. Age, sex, and the four other dimensions were included as covariates in all the models. Linkage-disequilibrium (LD) plots were designed using the LocusZoom software, based on 1000 genome CEU population data (hg19/1000 Genomes Mar 2012 EUR)^43^. For SNP annotation, we used the Infinium PsychArray Gene Annotation File provided by Illumina (https://support.illumina.com/downloads/infinium-psycharray-product-support-files.html).

Power calculations were performed with Genetic Association Study Power Calculator software (http://csg.sph.umich.edu/abecasis/cats/gas_power_calculator/reference.html) to determine the power of our study to detect associations considering our sample size.

Gene-based association analyses were performed via the Sequence Kernel Association Test (SKAT)^44^ using the SKATMeta library^45^ for R software. This type of gene-based analysis has the advantage of including rare variants, which were not considered in SNP-based analyses given the lack of statistical power for detecting associations with these variants at a single-variant level. SKAT combines the effects of common and rare variants in gene-sets, increasing the power to detect small effects.

Only results from genes with at least two genotyped markers were considered. A false discovery rate (FDR) correction was used as a significance criterion.

Finally, enrichment analyses were performed for genes with at least two genotyped markers that had a SKAT *p*-value lower than 0.01 using web-accessible DAVID (Database for Annotation, Visualization, and Integrated Discovery) Bioinformatic Resources v6.8^46,47^. This software analyzes input genes in the context of a genomic background (in this study, we selected the entire human genome as background) to cluster genes enriched in biological pathways and gene ontologies. We ordered the reported results by the FDR statistic to prioritize for further interpretation.

## Results

### Subjects and genotyping quality control

Three hundred and seventy-six (*n* = 376) samples passed quality control procedures. Table 1 summarizes the sociodemographic and clinical data including DY-BOCS scores gathered for the final OCD sample.

**Table 1:** Sociodemographic and clinical characteristics of the sample of 376 OCD patients.

Age, years	35.2 ± 10.7 (18–71)
Male/Female	186/190 (49.5/ 50.5)
Age at onset of OCD	19.9 ± 8.9 (4–46)^a^
*Y-BOCS score*	
Global	25.8 ± 5.5 (9–40)
Obsessions	12.6 ± 3.6 (0–20)
Compulsions	12.3 ± 4.0 (0–20)
*Baseline HDRS score*	12.2 ± 6.0 (0–29)
*Current comorbidity*	
No comorbidity	212 (56.4)
Mood disorder	71 (18.9)
Tics	52 (13.8)
Eating disorders	19 (05.1)
*Presence of dimensions in worst-ever period*	
Aggresive/checking	278 (73.9)
Symmetry/ordering	162 (43.1)
Contamination/cleaning	172 (45.7)
Hoarding	91 (24.2)
Sexual/religious	95 (25.3)
*Family psychiatric history*	
No psychiatric diagnosis	138 (36.7)
OCD	81 (21.5)
Mood disorder	114 (30.3)
Tics/ Tourette sydrome	35 (9.3)

Data are mean ± SD (range) or percentage (%).

*OCD* obsessive compulsive disorder, *Y-BOCS* Yale-Brown Obsessive Compulsive Scale, *HDRS* Hamilton Depression Rating Scale.

^a^Age at onset was collected for 374 patients (*n* = 374).

### SNP-level association analyses

Our total dataset consisted of 338,357 autosomal markers, of which 258,937 were SNPs (MAF ≥ 0.05).

**Fig. 1:**
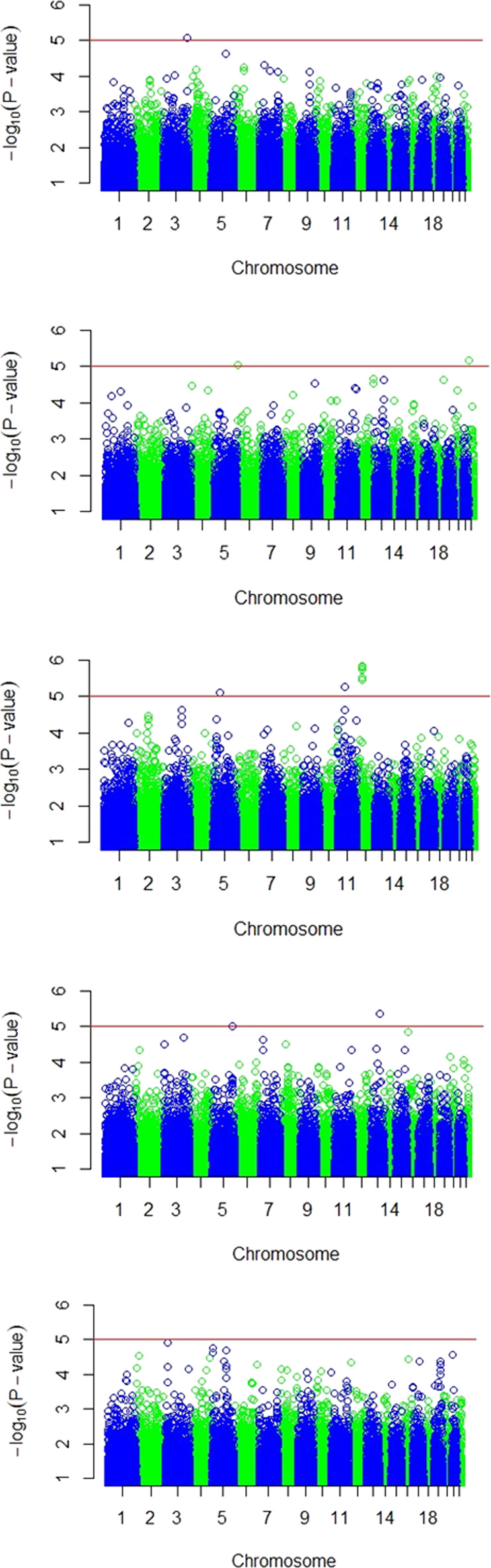
Manhattan plots for the genome-wide association analyses of genetic variations and OCD dimensions. **a** Aggressive dimension. **b** Contamination dimension. **c** Order dimension. **d** Hoarding dimension. **e** Sexual/religious dimension. A blue line indicates the level of suggestive evidence of association (*p* < 1 × 10^−5^).

No SNP exceeded the statistical threshold for genome-wide significance (*p* < 5 × 10^−8^) in any dimension (Fig. 1). The results at a *p* value ≤ 10^−4^ for the five dimensions can be seen in Table 2. Suggestive associations (*p* < 10^−5^) were found with the aggressive, contamination, order, and hoarding dimensions (Fig. 1a–d).

**Table 2:** Results from SNP_level regression analyses on OCD dimensions.

SNP	N Cases (MAF)	N Controls (MAF)	OR (CI)	*P*	CHR	Position (BP)	A1/A2	Gene	Upstream gene	Downstream gene
*Aggressive dimension*										
rs11127905	277 (0.28)	97 (0.14)	2.55 (2.01–3.25)	9.89 × 10^−05^	chr3	85710336	[T/C]	*CADM2*		
rs1202392	278 (0.13)	97 (0.25)	0.42 (0.34–0.52)	7.91 × 10^−05^	chr7	148914740	[A/G]	*ZNF282*		
rs13064299	278 (0.43)	97 (0.63)	0.45 (0.37–0.54)	8.66 × 10^−06^	chr3	187865100	[T/C]		*RP11-430L16.1* (292)	*LPP-AS2* (3894)
rs13180857	278 (0.37)	97 (0.54)	0.45 (0.37–0.54)	2.31 × 10^−05^	chr5	124754302	[T/C]		*RP11-395P13.6* (22629)	*RP11-756H20.1* (74652)
rs16918167	278 (0.27)	97 (0.43)	0.49 (0.41–0.59)	7.96 × 10^−05^	chr9	101815339	[T/G]	*COL15A1*		
rs2189993	278 (0.54)	97 (0.38)	2.13 (1.76–2.57)	7.10 × 10^−05^	chr7	82193853	[A/G]		*CACNA2D1* (120739)	*MTHFD2P5* (25311)
rs3799867	278 (0.14)	97 (0.27)	0.42 (0.33–0.52)	5.62 × 10^−05^	chr6	46594121	[A/G]	*CYP39A1*		
rs41307	278 (0.45)	97 (0.62)	0.47 (0.39–0.57)	4.95 × 10^−05^	chr7	28674854	[T/C]	*CRX10B5*		
rs6819000	278 (0.34)	97 (0.50)	0.48 (0.40–0.57)	6.46 × 10^−05^	chr4	65574910	[T/G]		*RP11-63H19.6* (97304)	*RP11-158O16.1* (57938)
rs9463221	278 (0.14)	97 (0.27)	0.42 (0.34–0.52)	7.22 × 10^−05^	chr6	46588027	[T/G]	*CYP39A1*		
*Contamination dimension*										
rs10466538	172 (0.18)	203 (0.08)	2.71 (2.13–3.46)	3.95 × 10^−05^	chr11	125169468	[T/G]	*PKNOX2*		
rs10882846	172 (0.15)	203 (0.26)	0.45 (0.37–0.55)	9.18 × 10^−05^	chr10	82984432	[T/C]		*AL356154.1* (79945)	*NRG3* (650638)
rs11016302	172 (0.20)	203 (0.09)	2.35 (1.89–2.92)	8.99 × 10^−05^	chr10	130320996	[A/C]		*RP11–264X1018.1* (202518)	*RP11-442O18.2* (390147)
rs11158195	172 (0.41)	203 (0.55)	0.54 (0.46–0.63)	8.80 × 10^−05^	chr14	58507598	[A/G]	*C14orf37*		
rs11210992	171 (0.37)	203 (0.52)	0.54 (0.46–0.63)	6.74 × 10^−05^	chr1	44878549	[T/C]	*RNF220*		
rs12832515	172 (0.32)	203 (0.19)	2.19 (1.82–2.63)	2.15 × 10^−05^	chr12	128219762	[A/G]		*RP11-526P6.1* (98750)	*RP11-749H20.1* (58310)
rs1557627	170 (0.32)	203 (0.18)	2.34 (1.94–2.83)	6.96 × 10^−06^	chr22	19677207	[T/C]		*AC000067.1* (22129)	*sx10p-05* (24780)
rs16935065	172 (0.22)	203 (0.11)	2.32 (1.88–2.86)	5.96 × 10^−05^	chr8	69699756	[A/G]	*C8orf34*		
rs2492501	172 (0.28)	203 (0.16)	2.10 (1.75–2.52)	4.98 × 10^−05^	chr1	112931256	[T/G]		*snoU13* (17527)	*CTTNBP2NL* (7547)
rs515077	172 (0.44)	203 (0.29)	2.07 (1.76–2.44)	9.00;× 10^−06^	chr6	8180286	[A/G]		*EEF1E1* (77475)	*RP11–203H2.1* (149602)
rs6477714	172 (0.30)	203 (0.45)	0.51 (0.44–0.60)	3.04 × 10^−05^	chr9	112367400	[T/G]		*YBX1P6* (70788)	*PALM2* (35668)
rs7671165	172 (0.20)	203 (0.33)	0.46 (0.39–0.56)	3.39 × 10^−05^	chr4	6401102	[T/C]	*PPP2R2C*		
rs78287729	172 (0.10)	203 (0.03)	4.35 (3.04–6.24)	4.53 × 10^−05^	chr4	145192851	[T/G]		*GYPA* (130947)	*RP11-361D14.2* (234201)
rs7925725	172 (0.33)	203 (0.48)	0.51 (0.43–0.60)	4.24 × 10^−05^	chr11	131449365	[A/C]	*NTM*		
rs7968104	172 (0.31)	203 (0.18)	2.16 (1.80–2.60)	2.97 × 10^−05^	chr12	128222410	[A/G]		*RP11-526P6.1* (101398)	*RP11-749H20.1* (55662)
rs8123755	172 (0.07)	203 (0.17)	0.34 (0.26–0.44)	4.63 × 10^−05^	chr20	23682133	[A/G]		*CST4* (12456)	*CST2P1* (9824)
rs930134	172 (0.57)	203 (0.42)	1.95 (1.67–2.29)	2.41 × 10^−05^	chr18	71568010	[A/G]		*RN7SL401P* (155546)	*RP11-25L3.3* (13630)
rs9546461	172 (0.57)	203 (0.42)	1.85 (1.58–2.16)	8.42 × 10^−05^	chr13	84212815	[T/C]		*RNU6-67P* (340513)	*SLITRK1* (238529)
rs985035	172 (0.57)	203 (0.41)	1.93 (1.65–2.25)	2.37 × 10^−05^	chr13	84262897	[A/G]		*RNU6-67P* (390595)	*SLITRK1* (188447)
*Order dimension*										
rs1039038	172 (0.46)	203 (0.51)	0.48 (0.41–0.57)	5.54 × 10^−06^	chr11	40511611	[A/C]	*LRRC4C*		
rs10771752	172 (0.49)	202 (0.47)	2.21 (1.87–2.61)	1.83 × 10^−06^	chr12	30802885	[A/C]	*IPO8*		
rs10979978	172 (0.25)	203 (0.34)	0.49 (0.41–0.59)	7.79 × 10^−05^	chr9	112406744	[T/C]	*PALM2*		
rs12146709	172 (0.23)	203 (0.20)	2.50 (2.05–3.05)	3.70 × 10^−06^	chr12	30888001	[T/C]	*CAPRIN2*		
rs12294573	172 (0.35)	203 (0.36)	1.97 (1.67–2.33)	4.75 × 10^−05^	chr11	24087714	[T/C]		*RNU6-783P* (216286)	*RP11-2F20.1* (169309)
rs14139	172 (0.50)	203 (0.47)	2.22 (1.88–2.63)	1.50 × 10^−06^	chr12	30782598	[T/C]	*IPO8*		
rs1601548	172 (0.39)	203 (0.38)	0.50 (0.42–0.59)	6.30 × 10^−05^	chr2	108735612	[T/C]		*AC023672.1* (19664)	*AC019100.3* (48592)
rs16888991	172 (0.29)	203 (0.30)	2.02 (1.70–2.40)	4.35 × 10^−05^	chr5	21168358	[T/C]		*RP11-774D14.1* (230558)	*RP11-811J10.1* (28471)
rs1811248	172 (0.33)	203 (0.38)	0.52 (0.44–0.61)	8.40 × 10^−05^	chr7	28421036	[T/G]	*CRX10B5*		
rs2249654	172 (0.19)	203 (0.18)	0.36 (0.29–0.46)	8.04 × 10^−06^	chr5	53892279	[A/G]		*SNX18* (49864)	*AC112198.1* (211759)
rs2724693	172 (0.29)	203 (0.27)	2.02 (1.71–2.39)	2.47 × 10^−05^	chr3	137807259	[A/G]	*DZIP1L*		
rs2724693	172 (0.29)	203 (0.27)	2.02 (1.71–2.39)	2.47 × 10^−05^	chr3	137807259	[A/G]	*DZIP1L*		
rs2724697	172 (0.29)	203 (0.27)	1.98 (1.68–2.34)	3.82 × 10^−05^	chr3	137798155	[T/C]	*DZIP1L*		
rs2993531	172 (0.41)	203 (0.44)	1.95 (1.65–2.30)	5.20 × 10^−05^	chr1	212190116	[A/C]	*INTS7*		
rs2993531	172 (0.41)	203 (0.44)	1.95 (1.65–2.30)	5.20 × 10^−05^	chr1	212190116	[A/C]	*INTS7*		
rs34473884	172 (0.17)	203 (0.20)	0.40 (0.32–0.50)	6.15 × 10^−05^	chr10	133761285	[A/G]	*PPP2R2D*		
rs442800	172 (0.29)	203 (0.26)	1.98 (1.67–2.34)	5.53 × 10^−05^	chr3	137786442	[T/C]	*DZIP1L*		
rs4963128	172 (0.33)	203 (0.26)	0.49 (0.41–0.59)	8.00 × 10^−05^	chr11	589564	[T/C]	*PHRF1*		
rs6487927	172 (0.50)	203 (0.47)	2.21 (1.87–2.61)	1.58 × 10^−06^	chr12	30826335	[T/C]	*IPO8*		
rs6487928	172 (0.23)	203 (0.20)	2.53 (2.07–3.08)	2.99 × 10^−06^	chr12	30828095	[A/G]	*IPO8*		
rs7316477	172 (0.50)	203 (0.47)	2.22 (1.88–2.63)	1.50 × 10^−06^	chr12	30786098	[A/G]	*IPO8*		
rs750523	172 (0.39)	203 (0.38)	0.49 (0.41–0.58)	3.40 × 10^−05^	chr2	108746221	[T/C]		*AC023672.1* (30273)	*AC019100.3* (37983)
rs756474	172 (0.23)	203 (0.32)	0.47 (0.39–0.57)	6.73 × 10^−05^	chr8	95634950	[A/G]		*RP11-267M23.7* (28121)	*RP11-22C11.2* (14563)
rs7937152	172 (0.41)	203 (0.39)	1.95 (1.65–2.29)	4.44 × 10^−05^	chr11	131936494	[T/C]	*NTM*		
rs7944117	172 (0.39)	203 (0.41)	1.93 (1.65–2.26)	2.44 × 10^−05^	chr11	40591800	[T/C]	*LRRC4C*		
rs832665	171 (0.47)	203 (0.46)	0.50 (0.43–0.59)	4.22 × 10^−05^	chr2	108731604	[T/C]		*AC023672.1* (15656)	*AC019100.3* (52600)
rs894943	172 (0.26)	203 (0.27)	0.49 (0.40–0.58)	8.80 × 10^−05^	chr17	74916307	[A/G]	*MGAT5B*		
*Hoarding dimension*										
rs1010156	91 (0.62)	284 (0.45)	2.22 (1.83–2.69)	3.19 × 10^−05^	chr8	23190941	[T/C]	*LOXL2*		
rs10187022	91 (0.49)	284 (0.33)	2.13 (1.77–2.56)	4.71 × 10^−05^	chr2	34341570	[A/C]	*AC009499.1*		
rs12670403	91 (0.58)	284 (0.41)	2.35 (1.92–2.88)	2.37 × 10^−05^	chr7	17309279	[A/C]		*AC098592.8* (236177)	*AC003075.4* (10179)
rs13068223	91 (0.35)	284 (0.52)	0.44 (0.36–0.53)	2.13 × 10^−05^	chr3	156470955	[A/G]	*LINC00886*		
rs1469064	91 (0.49)	284 (0.32)	2.39 (1.97–2.92)	9.95 × 10^−06^	chr5	164859465	[T/C]	*CTC-535M15.2*		
rs17137472	91 (0.58)	284 (0.41)	2.26 (1.85–2.76)	4.71 × 10^−05^	chr7	17296072	[T/C]		*AC098592.8* (222970)	*AC003075.4* (23386)
rs1807512	90 (0.46)	282 (0.31)	2.16 (1.78–2.63)	9.10 × 10^−05^	chr22	17221495	[T/C]		*VWFP1* (36128)	*AC005301.8* (6264)
rs1944828	91 (0.21)	284 (0.10)	2.92 (2.25–3.80)	4.53 × 10^−05^	chr11	127048359	[T/C]		*CTD-2234N14.2* (38729)	*CTD-2234N14.1* (12305)
rs285714	91 (0.40)	284 (0.26)	2.24 (1.84–2.73)	4.67 × 10^−05^	chr15	93126781	[T/C]	*RP11–386M24.3*		
rs4143121	91 (0.45)	284 (0.31)	2.29 (1.88–2.80)	3.16 × 10^−05^	chr3	1764453	[T/C]		*AC090043.1* (126524)	*RPL23AP39* (7301)
rs7326856	91 (0.43)	283 (0.26)	2.12 (1.77–2.55)	4.16 × 10^−05^	chr13	66338922	[T/C]		*STARP1* (453837)	*HNRNPA3P5* (23142)
rs956818	91 (0.18)	284 (0.35)	0.37 (0.30–0.47)	1.41 × 10^−05^	chr16	6537353	[T/G]	*RP11-420N3.2*		
rs956818	91 (0.18)	284 (0.35)	0.37 (0.30–0.47)	1.41 × 10^−05^	chr16	6537353	[T/G]	*RBFOX1*		
rs9575590	91 (0.33)	284 (0.52)	0.42 (0.35–0.51)	4.47 × 10^−06^	chr13	85066303	[A/G]		*UBE2D3P4* (471406)	*MTND4P1* (27935)
*Sexual/ religious dimension*										
rs10076327	95 (0.44)	280 (0.27)	2.41 (1.96–2.97)	2.12 × 10^−05^	chr5	122367347	[A/G]	*PPIC*		
rs10408163	95 (0.41)	279 (0.26)	2.24 (1.83–2.73)	5.49 × 10^−05^	chr19	47597102	[T/C]	*ZC3H4*		
rs10804925	95 (0.55)	280 (0.39)	2.11 (1.75–2.55)	7.39 × 10^−05^	chr3	190094024	[A/G]	*CLDN16*		
rs10902225	95 (0.28)	280 (0.45)	0.47 (0.39–0.57)	9.14 × 10^−05^	chr11	835065	[T/C]	*CD151*		
rs11128702	95 (0.33)	280 (0.20)	2.33 (1.89–2.88)	6.31 × 10^−05^	chr3	14530133	[T/C]	*SLC6A6*		
rs11131820	95 (0.46)	280 (0.29)	2.16 (1.79–2.60)	3.48 × 10^−05^	chr4	178683325	[T/C]	*LINC01098*		
rs12213157	95 (0.32)	280 (0.18)	2.31 (1.88–2.84)	5.22 × 10^−05^	chr6	163640489	[A/G]	*PACRG*		
rs12552120	95 (0.36)	280 (0.51)	0.47 (0.38–0.57)	8.87 × 10^−05^	chr9	90514482	[T/C]		*SPATA31E1* (10668)	*SPATA31C1* (14926)
rs12929114	95 (0.55)	280 (0.37)	2.18 (1.80–2.63)	3.79 × 10^−05^	chr16	65532244	[T/C]	*LINC00922*		
rs1441537	95 (0.11)	280 (0.24)	0.32 (0.25–0.42)	2.34 × 10^−05^	chr5	3261155	[T/C]		*CTD-2029E14.1* (79809)	*LINC01019* (156111)
rs144597	94 (0.46)	280 (0.29)	2.17 (1.79–2.64)	6.90 × 10^−05^	chr5	122223232	[A/C]	*SNX24*		
rs1460040	95 (0.42)	280 (0.26)	2.30 (1.87–2.82)	5.59 × 10^−05^	chr5	122105232	[A/G]		*RP11-166A12.1* (38852)	*SNX2* (5459)
rs1606502	95 (0.58)	280 (0.44)	2.13 (1.76–2.58)	7.95 × 10^−05^	chr8	57529208	[T/C]		*RP11-17A4.1* (27862)	*RP11-17A4.4* (23252)
rs16871518	95 (0.14)	280 (0.29)	0.35 (0.28–0.45)	1.76 × 10^−05^	chr5	3254413	[T/C]		*CTD-2029E14.1* (73067)	*LINC01019* (162853)
rs17023204	95 (0.28)	280 (0.17)	2.43 (1.94–3.04)	7.53 × 10^−05^	chr4	148329646	[T/C]		*MIR548G* (63777)	*RP11–364M6.1* (17480)
rs17635830	95 (0.09)	280 (0.24)	0.32 (0.24–0.42)	4.30 × 10^−05^	chr17	52847941	[T/C]		*ARL2BPP8* (15931)	*RN7SKP14* (369)
rs2303108	95 (0.39)	280 (0.25)	2.23 (1.82–2.73)	7.46 × 10^−05^	chr19	47589895	[T/C]	*ZC3H4*		
rs2303108	95 (0.39)	280 (0.25)	2.23 (1.82–2.73)	7.46 × 10^−05^	chr19	47589895	[T/C]	*ZC3H4*		
rs2345493	95 (0.14)	280 (0.29)	0.37 (0.29–0.47)	2.95 × 10^−05^	chr2	18292201	[T/G]	*KCNS3*		
rs2826856	95 (0.29)	280 (0.15)	2.42 (1.96–2.99)	2.67 × 10^−05^	chr21	22842175	[A/G]	*NCAM2*		
rs35521	95 (0.36)	280 (0.20)	2.33 (1.90–2.87)	4.29 × 10^−05^	chr5	107081478	[A/G]		*RN7SL782P* (10711)	*RN7SKP122* (64852)
rs3844159	95 (0.26)	280 (0.13)	2.43 (1.94–3.04)	8.07 × 10^−05^	chr10	60733376	[T/C]		*RN7SKP196* (104088)	*LINC00844* (26010)
rs4685147	95 (0.62)	280 (0.44)	2.30 (1.90–2.78)	1.23 × 10^−05^	chr3	14424656	[T/C]		*RP11–536I6.1* (30588)	*RNA5SP124* (11492)
rs4853954	95 (0.49)	280 (0.32)	2.06 (1.72–2.47)	6.58 × 10^−05^	chr2	2935910	[T/C]	*AC019118.2*		
rs62136856	95 (0.41)	280 (0.26)	2.26 (1.85–2.76)	4.17 × 10^−05^	chr19	47573527	[A/G]	*ZC3H4*		
rs7470248	95 (0.36)	280 (0.51)	0.47 (0.38–0.57)	8.87 × 10^−05^	chr9	90515619	[A/G]		*SPATA31E1* (11805)	*SPATA31C1* (13789)
rs974728	95 (0.21)	278 (0.36)	0.41 (0.33–0.51)	4.59 × 10^−05^	chr12	11934852	[A/G]	*X10TV6*		

*SNP* single nucleotide polymorphism, *MAF* minor allele frequency, *OR (CI)*, odds ratio (confidence interval); *A1/A2* allele 1, allele 2, *CHR*. chromosome, *BP* base pairs.

**Fig. 2:**
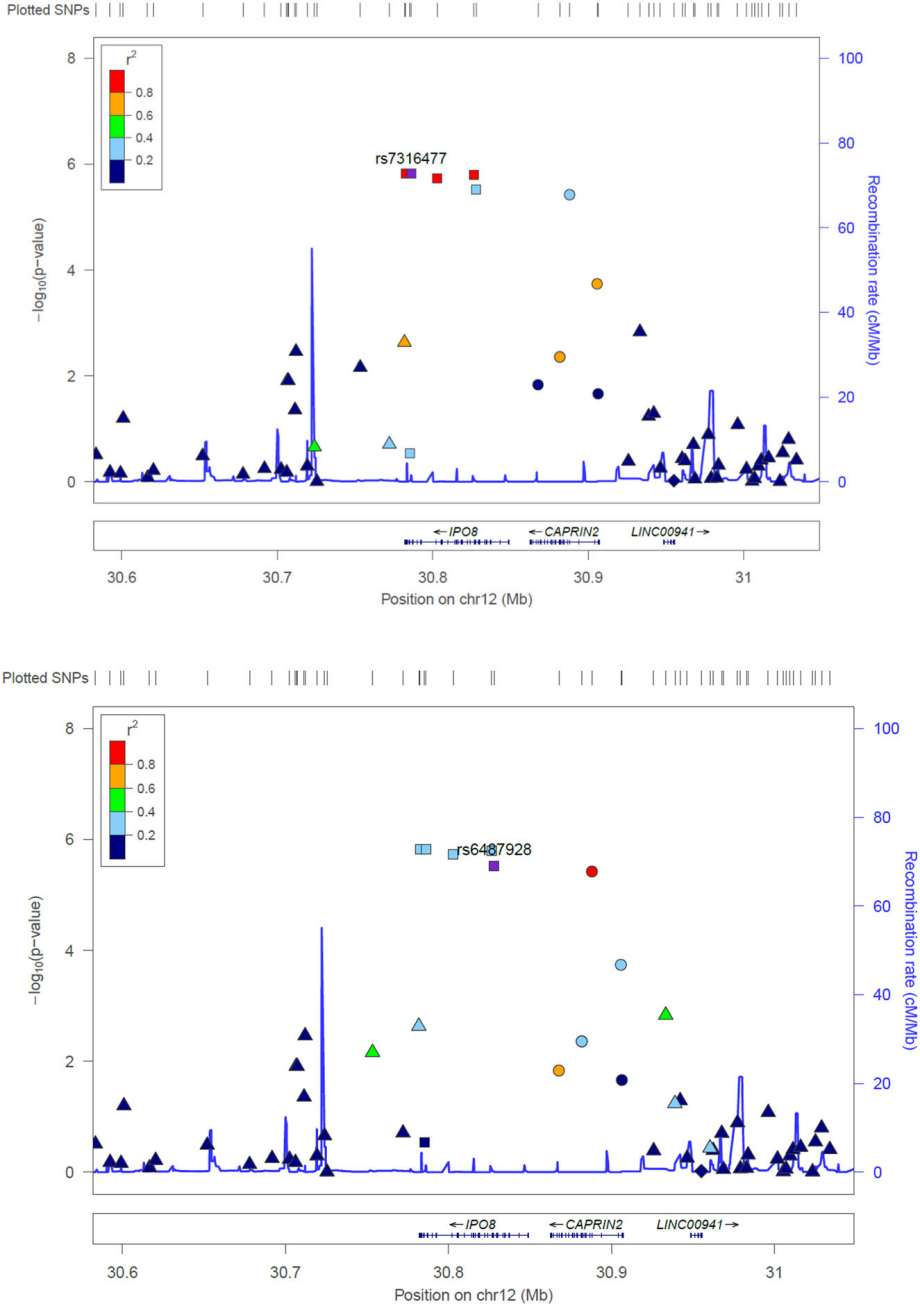
Regional association plots with LD reported for Order dimension-suggestively associated (*p* < 10^-5^) single-nucleotide polymorphisms (SNPs) in chromosome 12. **a** Plot of rs7316477. **b** Plot of rs6487928.

In the order dimension, eight variants presented suggestive associations (*p* < 10^−5^). Six of these variants were within chromosome 12 and, given the proximity between them (5 of them were variants of the *IPO8* gene), we performed LD analysis on this region. We found two clusters of SNPs: (1) rs7316477 (1.50 × 10^−6^), rs14139 (1.50 × 10^−6^), rs6487927 (1.58 × 10^−6^) and rs10771752 (1.83 × 10^−6^), all exonic variants located in the *IPO8* (Importin 8) gene (Fig. 2a); and (2) rs6487928 (2.99 × 10^−6^) and rs12146709 (3.70 × 10^−6^), exonic variants located in *IPO8* and *CAPRIN2* (Caprin Family Member 2), respectively (Fig. 2b). All these markers should be considered as forming a single association peak, since the level of association was not maintained for any of them when adjusting the model for each of the other markers.

### Gene-based association analyses

Gene-based analysis were performed for 22,017 (aggressive), 21,179 (order), 20,466 (contamination) and 22,472 (hoarding and sexual/religious) genes. Of these, 17,001 (aggressive), 16,467 (order), 15,790 (contamination), and 17,378 (hoarding and sexual/religious) had at least two genotyped markers and were considered in our further analyses.

Our results for genes with at least a suggestive association (*p* < 10^−4^) in the five dimensions can be seen in Table 3. One gene reached genome-wide significant association with hoarding (SET Domain Containing 3, Actin Histidine Methyltransferase, *SETD3*; *p* = 1.89 × 10^−08^). This gene codes for a protein involved, among other functions, in actin binding and modification, histone methylation, chromatin organization, and regulation of transcription^48^. The second most significant gene was *CPE* (Carboxypeptidase E), which reached genome-wide significant association with the aggressive dimension (*p* = 4.42 × 10^−6^). This gene codes for a membrane protein involved in the synthesis of peptide hormones and neurotransmitters as well as different neurotrophic functions^49^.

**Table 3:** Best gene-based results from SKAT analyses for the different OCD dimensions.

Gene	*P*	QMETA	CMAF	NSNPS
Aggressive				
* CPE*	**4.42** × **10**^**−06**^	10 857.8	4.18	15
* HIST1H2AH*	5.13 × 10^−05^	9 329.72	0.03	2
* FOXP4*	1.01 × 10^−05^	11 790.03	1.48	8
* CREB5*	9.29 × 10^−05^	6 440.17	2.87	12
Order				
* PPP2R2D*	6.54 × 10^−05^	23.49	0.65	3
Hoarding				
* SERINC2*	8.17 × 10^−05^	10 941.25	0.26	9
* SETD3*	**1.89** × **10**^**−08**^	16 579.98	0.64	3
Sexual/Religious				
* CDC42BPA*	4.97 × 10^−05^	7 002.15	6.61	27
* GPR137B*	3.65 × 10^−05^	18 669.76	2.24	15
* LYZL4*	3.48 × 10^−05^	9 965.91	0.07	3
* ABR*	9.13 × 10^−05^	7 283.78	1.81	7

*CMAF* collected minor allele fruequency, *QMETA* test score reported by SKAT. Bold text highlights significant associations at a gene-based analysis level.

### Functional annotation

Functional annotation was gathered for a final set of 154 (aggressive), 103 (contamination), 111 (order), 196 (hoarding) and 167 (sexual/religious) genes. The threshold used to select the genes included in the enrichment analyses (*p* < 0.01) included 1% of the genes in all the dimensions except the hoarding dimension, for which 2% of the genes presented a *p*-value lower than 0.01.

Detailed information on the results of these analyses are given in Tables 4 and 5. In the case of the hoarding dimension, the different pathways obtained are clustered in two groups according to their biological similarity.

**Table 4:** Results from enrichment analyses on Aggressive, Order and Sexual/religious dimensions.

Category	Term	Count (%)	*P*-value	Genes	List total	Fold enrichment	FDR
*Aggressive*							
GOTERM_BP_DIRECT	GO:0010043~response to zinc ion	5 (3.29)	1.52 × 10^−04^	ALAD, ASS1, SLC30A8, CPS1, SLC30A6	128	18.22	0.002
REACTOME_PATHWAY	R-HSA-390918 (*Peroxisomal lipid metabolism*)	3 (1.97)	0.002	MLYCD, NUDT7, ACBD4	72	47.27	0.020
*Order*							
KEGG_PATHWAY	hsa04071:Sphingolipid signaling pathway	5 (4.63)	0.002	BID, CERS6, BDKRB2, PPP2R2D, ASAH1	33	8.69	0.025
*Sexual/religious*							
REACTOME_PATHWAY	R-HSA-416482 (*G alpha (12/13) signalling events)*	5 (3.03)	0.003	ABR, RAC1, SOS2, ADRA1A, GNB5	69	8.54	0.032

*Count* number of OCD genes involved in the pathway/ biological process, *List total* total number of genes involved in the pathway/process, *FDR* false discovery rate.

**Table 5:** Results from enrichment analyses on Hoarding dimension.

Annotation Cluster 1	Enrichment Score: 6.97						
Category	Term	Count (%)	*P*-value	Genes	List total	Fold enrichment	FDR
GOTERM_BP_DIRECT	GO:2001030~negative regulation of cellular glucuronidation	8 (3.90)	9.39 × 10^−14^	UGT1A7, UGT1A10, UGT1A6, UGT1A9, UGT1A8, UGT1A3, UGT1A4, UGT1A1	176	95.41	1.74 × 10^−14^
GOTERM_BP_DIRECT	GO:1904224~negative regulation of glucuronosyltransferase activity	8 (3.90)	9.39 × 10^−14^	UGT1A7, UGT1A10, UGT1A6, UGT1A9, UGT1A8, UGT1A3, UGT1A4, UGT1A1	176	95.41	1.47 × 10^−12^
GOTERM_BP_DIRECT	GO:0045922~negative regulation of fatty acid metabolic process	8 (3.90)	4.19 × 10^−13^	UGT1A7, UGT1A10, UGT1A6, UGT1A9, UGT1A8, UGT1A3, UGT1A4, UGT1A1	176	84.81	6.57 × 10^−12^
GOTERM_BP_DIRECT	GO:0052695~cellular glucuronidation	8 (3.90)	1.25 × 10^−10^	UGT1A7, UGT1A10, UGT1A6, UGT1A9, UGT1A8, UGT1A3, UGT1A4, UGT1A1	176	47.70	1.96 × 10^−09^
KEGG_PATHWAY	hsa00053:Ascorbate and aldarate metabolism	9 (4.39)	2.86 × 10^−10^	UGT1A7, UGT1A10, UGT1A6, UGT1A9, UGT1A8, UGT1A3, UGT1A5, UGT1A4, UGT1A1	77	29.78	3.33 × 10^−09^
REACTOME_PATHWAY	R-HSA-156588 (*Glucuronidation*)	8 (3.90)	5.67 × 10^−10^	UGT1A7, UGT1A6, UGT1A9, UGT1A8, UGT1A3, UGT1A5, UGT1A4, UGT1A1	104	38.78	7.06 × 10^−09^
KEGG_PATHWAY	hsa00040:Pentose and glucuronate interconversions	9 (4.39)	1.69 × 10^−09^	UGT1A7, UGT1A10, UGT1A6, UGT1A9, UGT1A8, UGT1A3, UGT1A5, UGT1A4, UGT1A1	77	24.36	1.97 × 10^−08^
KEGG_PATHWAY	hsa00140:Steroid hormone biosynthesis	10 (4.88)	1.04 × 10^−08^	UGT1A7, UGT1A10, UGT1A6, UGT1A9, UGT1A8, UGT1A3, UGT1A5, UGT1A4, UGT1A1, CYP19A1	77	15.40	1.21 × 10^−07^
KEGG_PATHWAY	hsa00860:Porphyrin and chlorophyll metabolism	9 (4.39)	1.33 × 10^−08^	UGT1A7, UGT1A10, UGT1A6, UGT1A9, UGT1A8, UGT1A3, UGT1A5, UGT1A4, UGT1A1	77	19.14	1.55 × 10^−07^
KEGG_PATHWAY	hsa00830:Retinol metabolism	9 (4.39)	4.11 × 10^−07^	UGT1A7, UGT1A10, UGT1A6, UGT1A9, UGT1A8, UGT1A3, UGT1A5, UGT1A4, UGT1A1	77	12.56	4.78 × 10^−06^
GOTERM_BP_DIRECT	GO:0042573~retinoic acid metabolic process	5 (2.44)	1.05 × 10^−05^	UGT1A7, UGT1A9, UGT1A8, UGT1A3, UGT1A1	176	34.07	1.65 × 10^−4^
GOTERM_BP_DIRECT	GO:0008152~metabolic process	11 (5.37)	1.06 × 10^−05^	UGT1A7, UGT1A10, UGT1A6, ACSM1, UGT1A9, UGT1A8, UGT1A3, UGT1A5, UGT1A4, GALNS, UGT1A1	176	6.25	1.67 × 10^−4^

Annotation Cluster 2	Enrichment Score: 2.96						
Category	Term	Count	*P-value*	Genes	List total	Fold enrichment	FDR
GOTERM_BP_DIRECT	GO:0019532~oxalate transport	4 (1.95)	1.73 × 10^−04^	SLC26A6, SLC26A5, SLC26A10, SLC26A2	176	34.69	0.003
GOTERM_BP_DIRECT	GO:1902358~sulfate transmembrane transport	4 (1.95)	2.28 × 10^−04^	SLC26A6, SLC26A5, SLC26A10, SLC26A2	176	31.80	0.004
GOTERM_BP_DIRECT	GO:0015701~bicarbonate transport	5 (2.44)	0.001	SLC26A6, SLC26A5, CFTR, SLC26A10, SLC26A2	176	10.84	0.017

*Count* number of OCD genes involved in the pathway/ biological process, *List total* total number of genes involved in the pathway/process; FDR, false discovery rate.

## Discussion

In this study, we examined the genomic bases of each of the most consistently validated symptom dimensions of OCD. Differential findings were obtained for the five dimensions considered. At the SNP-level, no variant reached genome-wide significance. The top SNPs were six markers in the order dimension (forming a single association peak) and one variant in the hoarding dimension (*p* < 5 × 10^−6^). Gene analyses showed one gene associated with hoarding (*SETD3*, *p* = 1.89 × 10^−8^) and another with the aggressive dimension (*CPE*, *p* = 4.42 × 10^−6^) at a genome-wide level. Different pathways or biological processes were represented by the aggressive, order, sexual/religious, and hoarding dimensions. For contamination, no pathway remained associated after FDR correction.

Six of the top variants at the SNP-level analysis conformed a genomic region involving *IPO8* and *CAPRIN2*, which presented an association signal with the order dimension. Certain intergenic variants near *CAPRIN2* presented association signals (*p* < 5 × 10^−5^) with different neuropsychological variables and personality traits (https://www.ncbi.nlm.nih.gov/projects/gap/cgi-bin/study.cgi?study_id=phs000342.v18.p11; https://www.ncbi.nlm.nih.gov/projects/gap/cgi-bin/study.cgi?study_id=phs000338.v1.p1). Considering the LD findings for this region and the fact that the six variants represent a single peak of association, it may be interesting to consider this genomic region in further studies, since it could be a relevant for the order dimension.

Gene-based analyses reported one gene associated with hoarding at the genome-wide significance level (*SETD3*, *p* = 1.89 × 10^−8^). That gene is expressed in brain regions associated with OCD^50,51^ such as caudate and cerebellum (The Human Protein Atlas, *SETD3*, gene available from https://www.proteinatlas.org/ENSG00000183576-SETD3/tissue) and seems to mediate transcriptome changes in the hypothalamus of mice^52^. It has also been associated with apoptotic processes, which in turn have been observed to mediate the neuronal loss in certain brain regions of BD patients^53^. Although two SNPs located near *SETD3* show suggestive associations with autoimmune and inflammatory conditions (rs2614463, *P* = 7.00 × 10^−6^; rs2664299, *P* = 9.00 × 10^−6^), none of them are in LD with any of the *SETD3* variants in our analysis—rs12886549 (*P* = 0.68, MAF = 0.24, OR = 1.09); rs8015827 (*P* = 0.79; MAF = 0.39; OR = 1.05); rs34322735 (*P* = 1.72 × 10-08, MAF = 0.01, OR = 101.39). *CPE*, which was associated with the aggressive dimension, is a gene highly expressed in brain^54^. Different polymorphisms in this gene have been associated with a loss of neuroprotective function by reducing the effects of the oxidative stress in human cell lines^55^ and transgenic mice^56^, leading to memory deficits and depressive behavior^56^. In addition, *CPE*-knockout mice have shown neurodegeneration in the hippocampus and the prefrontal cortex^57^.

In relation to the aggressive dimension, enrichment analyses showed overrepresentation in *response to zinc ions* (GO:0010043, FDR = 0.002). Zinc ions are highly prevalent in the brain, being especially prominent in forebrain glutamatergic neurons, the hippocampus, and the amygdala^58,59^, and they plays an important role in neuronal plasticity^60^. Zinc deficiency has been associated with cognitive decline, Alzheimer’s disease (AD) and different psychiatric disorders in the elderly. In addition, genetic variants within *ZNF142*, a gene coding for a zinc-finger protein, have been associated with a neurodevelopmental disorder resulting in speech impairment and intellectual disability^61^. The *Peroxisomal lipid metabolism* pathway (R-HSA-390918) was also significantly enriched in the aggressive dimension. Lipid metabolism has been extensively associated with different neuropsychiatric disorders, such as BD and depressive disorders, as well as AD^62–65^. Similarly, the *sphingolipid signaling pathway* (hsa04071) appears overrepresented in the order dimension. Sphingolipids are structural elements of cellular membranes and they play a role in cell signaling, differentiation and proliferation, apoptotic processes and inflammation^66^. Sphingolipid signaling has been observed to be involved in anxiety-like behavior in animal models as well as schizophrenia, depression and BD^66–69^.

Sexual/religious dimension genes were found enriched for *G alpha (12/13) signaling events* (R-HSA-416482). G12/13 subunits are alpha units of heterotrimeric G proteins that regulate different cell processes through the use of guanine nucleotide exchange factors (GEFs). This family of G-protein subunits has been associated with neurodevelopment and is involved in processes of cell proliferation and migration^70^. In addition, G12 subunits have been observed to influence memory consolidation and contextual retrieval in mice, via increased expression in the hippocampus^71^.

For the hoarding dimension, the first cluster of biological mechanisms and pathways includes cellular metabolic processes, such as lipid, vitamin and carbohydrate metabolism, all involving glucuronidation processes, as most genes included in these mechanisms or pathways code for UDP-glucuronosyltransferases (Table 5). It has been demonstrated that an alteration of the activity of these enzymes can affect brain function^72^. As an example, induction of UDP-glucuronosyltransferase 1A1 during the prenatal period can cause neurodevelopmental disorders in mice^73^.

There is increasing evidence from metabolomic studies of the importance of metabolic processes in psychiatric disorders. Post-traumatic stress disorder (PTSD) has been associated with the alteration of different kinds of metabolites, such as monosaccharides, nucleosides or fatty acids^74^. Furthermore, there is evidence of dysfunctional metabolism of lipids and vitamins in depressed patients^75^, which may explain the high prevalence of comorbid metabolic syndrome and cardiovascular disease^76^.

A lower plasma concentration of certain lipids with a neuroprotective role also has been observed in PTSD patients, compared to healthy controls^77^. Altered lipid metabolism has been found in other psychiatric conditions, such as MDD, BD or schizophrenia, and associated with symptoms including anxiety, stress and cognitive impairment^78^. Lipid metabolism in turn influences steroid synthesis, which has been associated with brain electrical activity through the role of lipids in modulating neuronal excitability^79^. In addition, an alteration of porphyrin and chlorophyll metabolism might affect the formation of heme groups, possibly leading to neurotoxic effects, among others^80,81^.

The second cluster of biological mechanisms and pathways for the hoarding dimension is related to anion transport. Most of the hoarding dimension genes involved are members of the solute-carrier 26 family A (SLC26A). These transporters have been observed to influence, among other functions, microbiome composition, pH regulation, and anion transport^82^, which in turn have been related with the pathophysiology of different psychiatric disorders^83^. More specifically, anion transport and pH regulation in the brain play a role in intra- and inter-signaling and plasticity processes^84^. In relation to microbiome composition, late-onset autism has been associated with differences in the gastrointestinal microflora when compared to healthy controls^85^. Moreover, exposure to certain microbial pathogens during fetal development has been associated with the pathogenesis of schizophrenia in humans^86^, and both anxiety-like behavior and cognitive impairment in rodents^87,88^. It is interesting to note the increase in the genetic weight observed in the hoarding dimension when the rare variants are included in the analysis, since only two variants at the SNP level reach suggestive association and no SNP reaches genome-wide significance. This contrasts with the findings obtained for this dimension in the gene-based and pathway analyses, which are notably more numerous than for the other dimensions. We think this suggests a role for rare variants in the hoarding dimension. We also believe that the consistently observed higher heritability of this dimension, compared to the others^9,10,89,90^, could mostly be explained by the influence of rare variants. Further research is needed to reveal the genetic bases of the hoarding dimension.

Although OCD symptom dimensions overcome the unitary clinical diagnosis of OCD, subtyping OCD according to overt clinical manifestations also presents significant limitations. Due to methodological differences, no concrete OCD dimensions classification system has been universally accepted. Some authors argue that other taxometric methods should be used to elucidate the symptom dimensions in OCD, including age of onset, comorbidities, or neuropsychological functioning in combination with clinical manifestations. The lack of significant associations among OCD symptom dimensions and individual SNPs could reflect limited statistical power due to the small sample size. Considering our total sample size, the number of cases and controls for each OCD dimension, a significance threshold of *p* < 2 × 10^−7^ (Bonferroni threshold for 258,000 SNPs analyzed) and a risk allele frequency (MAF) of 0.1, our study has 80% power to detect a relative risk (RR) of 3.5 and 5 (equivalent to OR = 4.8 and OR = 9) for order and sexual/religious dimensions, respectively; for the three other OCD dimensions, the detectable RR under these conditions is 4 (equivalent to OR = 6). A representation of the statistical power achieved for different MAF and RR thresholds is shown in Supplementary Fig. 1 (Fig. S1a–e). Furthermore, we would have liked to consider the severity score for each dimension in our analysis, in addition to their presence/absence, which would have been possible with a larger sample. However, our sample was thoroughly characterized phenotypically, and our results highlight important differences in relation to the genetic bases, as well as the genetic load of the different OCD dimensions. In addition, rare variants are considered at gene-based and pathway analyses, since this kind of analysis increases the power of detecting small effects. The inclusion of rare variants is important given the growing appreciation for their importance in neuropsychiatric disorders^91–93^.

OCD is a highly heterogeneous disorder in terms of symptom profile, comorbidity and underlying brain substrate, which represents a challenge for understanding and treating the disorder. This heterogeneity may confound and contribute to mostly negative findings in current genome-wide analysis studies, despite clear evidence for a strong genetic component of the disorder based on twin and family studies, ranging from 40–65%. Therefore, broad consensus has emerged for the need to explore OCD not as a homogeneous diagnosis, but rather considering other phenomenological approaches that investigate more refined phenotypes. In this sense, investigating genetic markers associated with different OCD symptom dimensions could be a useful strategy to begin disentangling the complex genetic vulnerability of the disorder. A clearer identification of susceptibility genes for OCD would translate into a better understanding of the etiology of the disorder and would help to develop potentially targeted and specific treatment approaches to improve the long-term outcome for OCD patients.

## Supplementary information

Supplementary Figure 1 (Figure S1)
