# Supplementary material for: Looking into the genetic bases of OCD dimensions: a pilot genome-wide association study
Source: Transl Psychiatry. 2020 May 18;10:151. doi: 10.1038/s41398-020-0804-z (PMC7235014; doi:10.1038/s41398-020-0804-z)
Supplement: Supplementary file 1 — Supplementary Figure 1 (Figure S1) [file 41398_2020_804_MOESM1_ESM.docx]

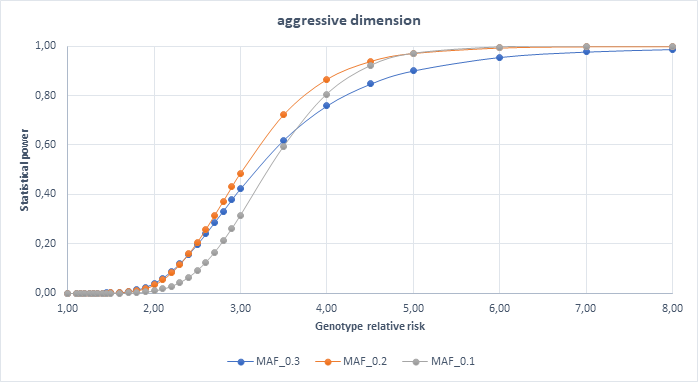


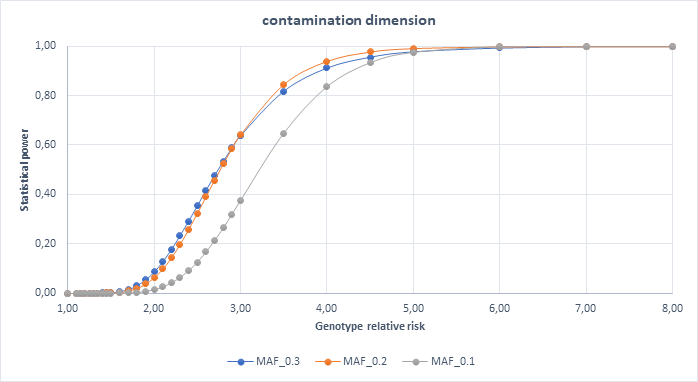

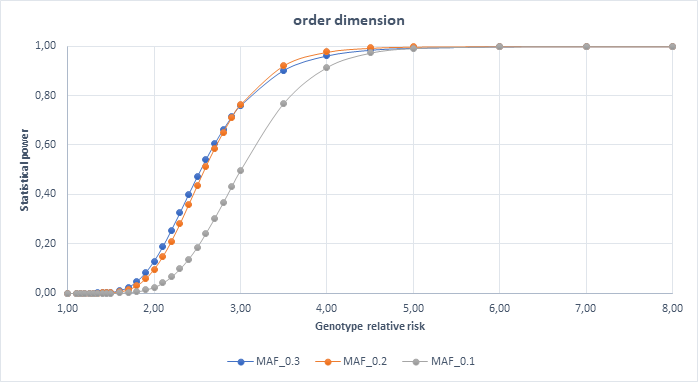

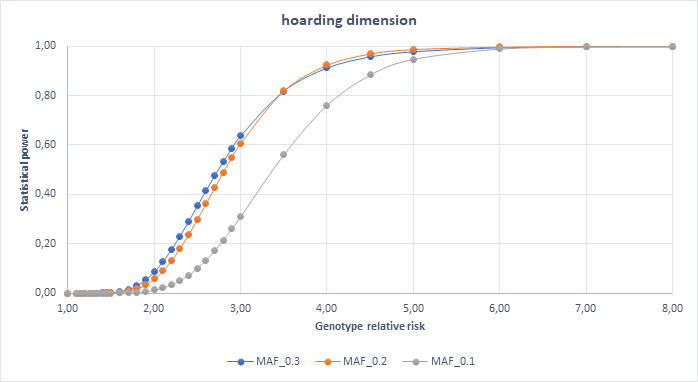

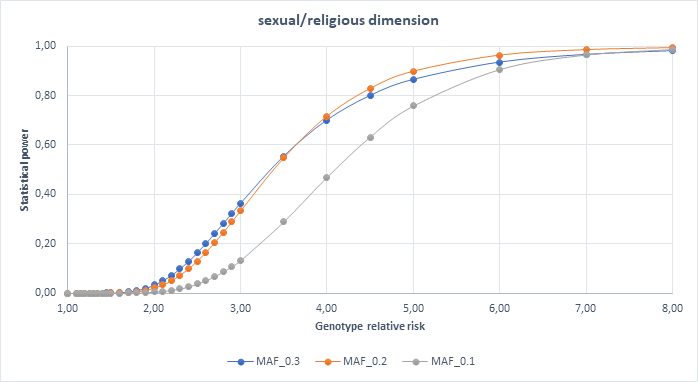


**Figure S1.** Statistical power plots for our study depending on the RR given three different MAF values. **(a)** Power plot for the aggressive dimension. **(b)** Contamination. **(c)** Order. **(d)** Hoarding. **(e)** The sexual/ religious dimension.
